# Deletion of Glut1 in early postnatal cartilage reprograms chondrocytes toward enhanced glutamine oxidation

**DOI:** 10.1038/s41413-021-00153-1

**Published:** 2021-08-23

**Authors:** Cuicui Wang, Jun Ying, Xiangfeng Niu, Xiaofei Li, Gary J. Patti, Jie Shen, Regis J. O’Keefe

**Affiliations:** 1 Department of Orthopaedic Surgery, School of Medicine, Washington University, St. Louis, MO USA; 2 Institute of Orthopaedics and Traumatology, The First Affiliated Hospital of Zhejiang Chinese Medical University, Hangzhou, China; 3 Zhejiang Chinese Medical University, Hangzhou, China; 4 Department of Chemistry, Genetics and Medicine, Washington University, St. Louis, MO USA

**Keywords:** Metabolism, Homeostasis

## Abstract

Glucose metabolism is fundamental for the functions of all tissues, including cartilage. Despite the emerging evidence related to glucose metabolism in the regulation of prenatal cartilage development, little is known about the role of glucose metabolism and its biochemical basis in postnatal cartilage growth and homeostasis. We show here that genetic deletion of the glucose transporter Glut1 in postnatal cartilage impairs cell proliferation and matrix production in growth plate (GPs) but paradoxically increases cartilage remnants in the metaphysis, resulting in shortening of long bones. On the other hand, articular cartilage (AC) with Glut1 deficiency presents diminished cellularity and loss of proteoglycans, which ultimately progress to cartilage fibrosis. Moreover, predisposition to Glut1 deficiency severely exacerbates injury-induced osteoarthritis. Regardless of the disparities in glucose metabolism between GP and AC chondrocytes under normal conditions, both types of chondrocytes demonstrate metabolic plasticity to enhance glutamine utilization and oxidation in the absence of glucose availability. However, uncontrolled glutamine flux causes collagen overmodification, thus affecting extracellular matrix remodeling in both cartilage compartments. These results uncover the pivotal and distinct roles of Glut1-mediated glucose metabolism in two of the postnatal cartilage compartments and link some cartilage abnormalities to altered glucose/glutamine metabolism.

## Introduction

Although they both originate from the embryonic common cartilage anlagen, the growth plate (GP) and articular cartilage (AC) separate and develop unique structures and functions shortly after birth as the primary and secondary ossification centers form and expand.^1^ During early postnatal development, GP chondrocytes are metabolically active and undergo a highly orchestrated sequence of events including cell proliferation followed by hypertrophic maturation, similar to the chondrocyte differentiation process that occurs during embryonic skeletogenesis.^2^ Normal functioning of GP chondrocytes determines longitudinal bone growth, and the cartilaginous extracellular matrix provides a template for future bone formation.^3^ Perturbation of the normal sequence of cartilage development and growth pre- and postnatally is known to cause a number of skeletal dysplasias in humans.^4^ In contrast to the case for GP, following embryonic joint formation and postnatal growth, the cellularity and phenotype of mature AC are maintained throughout adulthood via largely unknown mechanisms.^5^ AC chondrocytes secrete and maintain a highly organized extracellular matrix with extraordinary mechanical properties; however, they rarely proliferate and have limited capacity for cartilage regeneration.^6^ Disruption or impairment of the signals that are required to maintain these cells are thought to cause diseases in AC, such as osteoarthritis (OA).^7,8^

Although studies have defined unique molecular and signaling pathways and distinct regulatory networks that govern postnatal cartilage growth and homeostasis, the intrinsic character and nature of the chondrocytes in these two cartilage compartments remain to be fully elucidated. Glucose metabolism has been well recognized as a key regulatory hub frequently altered in many pathological conditions;^9–12^ however, much less is known about the physiological and pathological roles of glucose metabolism in cartilage. Chondrocytes, like most other mammalian cells, utilize glucose as a major energy source but also require glucose as the structural precursor for glycosaminoglycan synthesis. Early studies showed that GP and AC chondrocytes both favor glycolysis for energy production. Moreover, intrinsic metabolic differences are similarly exhibited by chondrocyte subpopulations within GPs and AC; with shifts toward more oxidative processes occurring in hypertrophic and deep-zone cells in GPs and AC, respectively.^13,14^ Nevertheless, it remains unclear whether the precise extents of glucose uptake and utilization vary between GP and AC chondrocytes overall. A prerequisite to answer this fundamental question is to define the biochemical basis of glucose metabolism in these cells.

More recently, studies from the Karsenty group^15^ and the Long group^16^ have revealed that Glut1-mediated glucose metabolism plays an essential role in embryonic cartilage development and long bone growth. Deletion of Glut1 in osteoprogenitors leads to reduced bone formation due to limited osteoblast differentiation and mineralization.^15^ Mice with Glut1 deficiency in mesenchymal cells exhibit impaired chondrocyte proliferation and hypertrophic maturation, leading to retarded skeletal development. Mechanistically, the BMP pathway has been shown to regulate GP chondrocyte Glut1 expression, glucose metabolism, and skeletal development.^16^ In addition to BMP2, other signaling pathways involved in glucose metabolism, such as the IGF pathway, can also affect cartilage development. Reports have indicated that knockout of IGF1R results in a shortened cartilage template with a hypertrophic zone that is decreased in size.^17,18^ Similarly, uncoordinated GP development and imbalanced glucose consumption have also been observed in Igf2-null mice.^19^ Thus, evidence indicates that perturbation of glucose metabolism in chondrocytes during embryonic development via targeting of either Glut1 or the signaling molecules involved in the regulation of glucose metabolism profoundly alters the course of chondrocyte maturation and limb formation processes, including cell proliferation and hypertrophy, and cartilage matrix production, suggesting a key role for glucose metabolism during endochondral ossification and cartilage development. On the other hand, metabolic changes in AC chondrocytes are also believed to occur in AC with OA.^20–23^ Given the molecular complexity of this emerging regulatory mechanism in cartilage, it is not known whether glucose metabolism is required for postnatal cartilage growth and homeostasis and, if so, whether it elicits unifying or distinct impacts on GP and AC through adulthood.

Here, through genetic and metabolic approaches, we found that Glut1-mediated glucose metabolism is required for normal GP and AC homeostasis during postnatal growth. Glut1 LOF led to decreased cell proliferation and matrix production but paradoxically increased the amounts of cartilage remnants in the metaphysis underneath the GP over time. On the other hand, Glut1 LOF AC displayed transient apoptosis, shifted toward a more catabolic state, and ultimately developed cartilage fibrosis. Mice with Glut1 LOF in the AC were less resistant to injury and developed accelerated OA. To compensate for reduced glucose uptake and utilization, chondrocytes shifted to glutamine oxidation as an alternative metabolic pathway. Despite the necessity for cell survival under conditions of glucose stress, uncontrolled glutamine flux may cause pathologies in extracellular matrix deposition and remodeling. Collectively, our data highlight glucose metabolism as a key factor in cartilage homeostasis and indicate that Glut1 LOF results in cartilage abnormalities in the AC and GP linked to altered energy metabolism and glutamine utilization.

## Results

### Glut1 is the primary isoform of glucose transporters in chondrocytes, and loss of Glut1 leads to diminished glucose metabolism

**Fig. 1:**
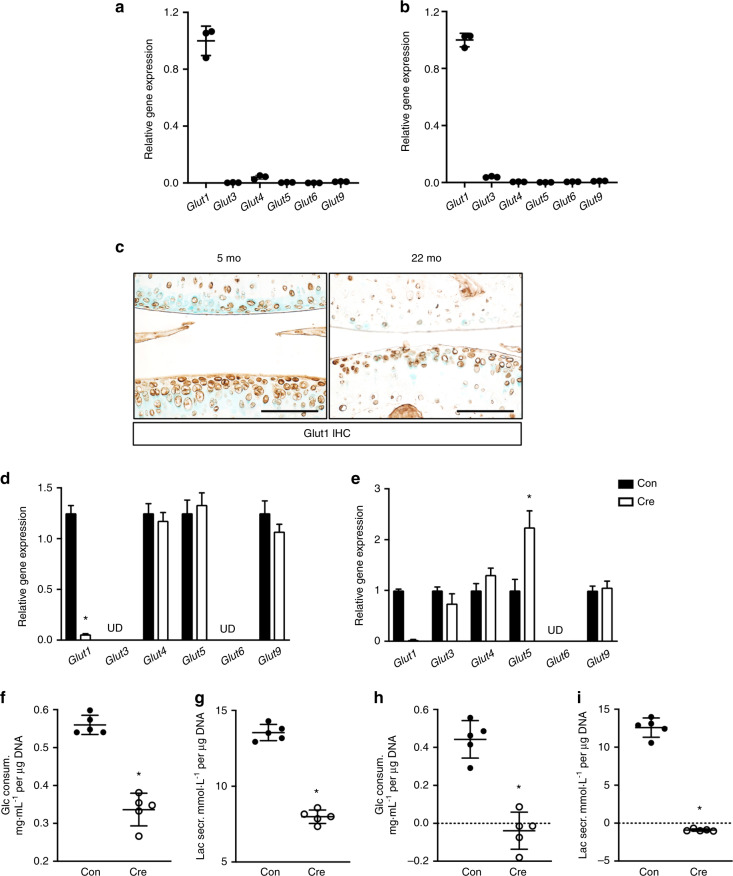
Glut1 encodes the primary glucose transporter in primary chondrocytes, and deletion of Glut1 leads to diminished glucose metabolism in chondrocytes. *N* = 3. **a**–**b** Gene expression of different glucose transporters in normal primary costal (GP) chondrocytes (**a**) and articular (AC) chondrocytes (**b**) as assayed by RT-qPCR. All mRNA abundances were normalized to that of *β-actin*. *N* = 3. *P < 0.05. **c** Immunostaining for Glut1 in knee sections of C57/BJ6 wild-type mice at 5 and 22 months. *N* = 3. Scale bar, 100 μm. **d**–**e** Gene expression of different glucose transporters in Ad-Con (control; Con)- or Ad-Cre (KO)-transduced *Glut1*^*f/f*^ primary GPs (**d**) and AC chondrocytes (**e**). The abundance of each individual mRNA in Glut1 KO cells was normalized to that in control cells. *N* = 3. *P < 0.05. **f**–**i** Glucose consumption and lactate secretion by control and Glut1 KO primary GP (**f**–**g**) and AC (**h**–**i**) chondrocytes over 24 h. The data were normalized to the genomic DNA content and are expressed as the mean ± SD. *N* = 5. *P < 0.05

To establish the main glucose transporters used by primary chondrocytes, we measured the relative gene expression of different glucose transporters that have been previously reported in human and murine chondrocytes.^24–26^ Consistent with previous findings, among the six genes examined in costal chondrocytes (referred to as GP chondrocytes hereafter) and AC chondrocytes, *Glut1* was expressed at markedly higher levels than other glucose transporters, suggesting that *Glut1* encodes the glucose transporter responsible for the majority of activity in both types of chondrocytes (Fig. 1a, b). Interestingly, comparison of AC tissues of 5- and 22-month-old mice revealed that Glut1 expression greatly decreased with aging (Fig. 1c). To determine the biological importance of Glut1 in primary chondrocytes, GP and AC chondrocytes were isolated from *Glut1*^*f/f*^ pups and virally transduced with Ad-Cre (or Ad-Con) to delete *Glut1* (Fig. 1d, e). None of the other Glut isoform transcripts showed compensatory increases in response to *Glut1* removal in either cell type except for Glut5, which showed a moderate elevation in expression in Glut1 LOF AC chondrocytes compared to its basal mRNA expression in control AC chondrocytes (Fig. 1e). Consequently, AC chondrocytes showed remarkable reductions in glucose consumption during a 24 h period (Fig. 1f). Likewise, lactate secretion was markedly decreased (Fig. 1g), reflecting impaired glucose uptake and glycolysis in Glut1 LOF AC chondrocytes. More strikingly, in the case of GP chondrocytes, removal of Glut1 led to nearly undetectable glucose consumption or lactate secretion (Fig. 1h, i), hence indicating complete blockade of glucose metabolism in these cells.

### Glut1 is required for postnatal GP growth

**Fig. 2:**
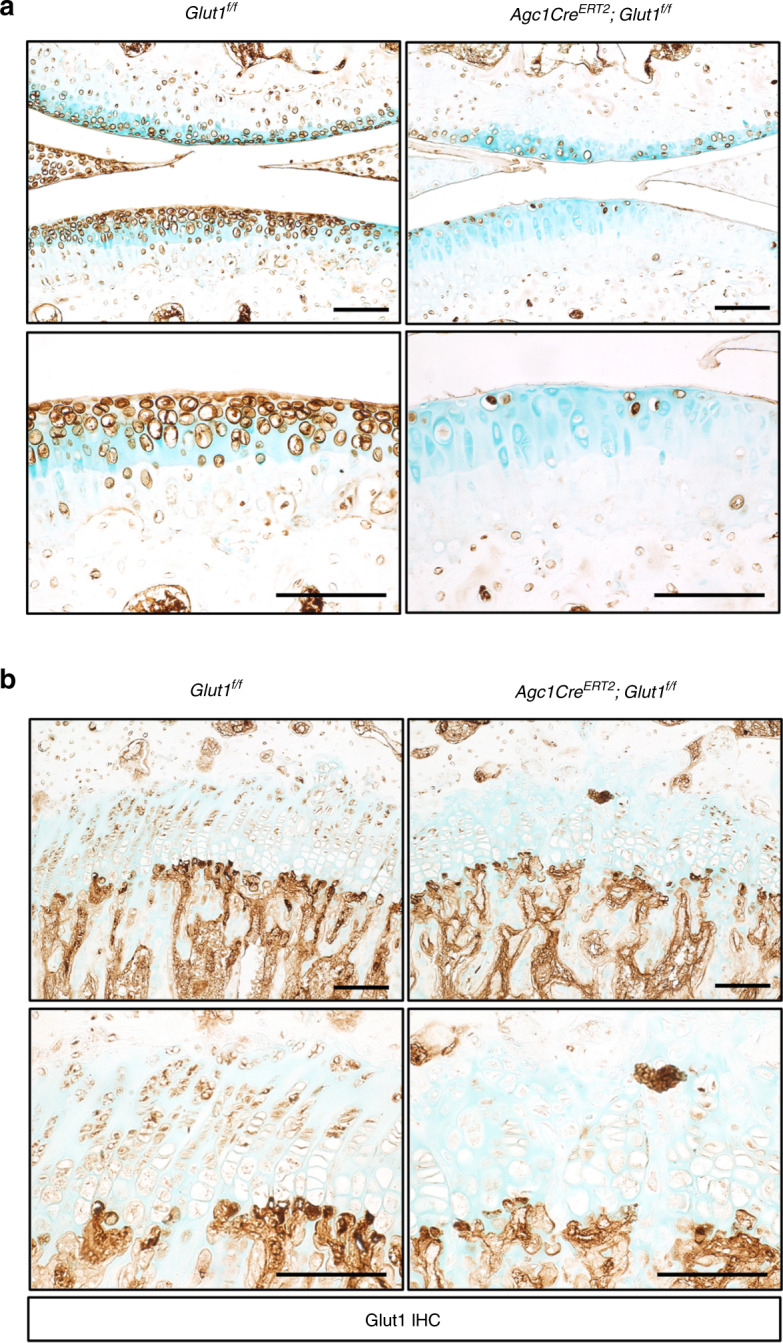
Postnatal genetic deletion with Agc1CreERT2 eliminates Glut1 in both the growth plate (GP) and articular cartilage (AC) in mouse long bones. **a**–**b** Immunostaining for Glut1 AC in knee sections (**a**) and GP tibia sections (**b**) of *Glut1*^*f/f*^ (control; Con) and *Agc1Cre*^*ERT2*^*;Glut1*^*f/f*^ (Glut1 LOF; Mut) mice 1 month following tamoxifen induction. *N* = 3. Scale bar, 100 μm

These observations prompted us to investigate whether Glut1-mediated glucose metabolism is required for postnatal GP growth and AC homeostasis. To answer this question, we first examined the cell linages that are targeted by *Agc1Cre*^*ERT2*^ by analyzing the cartilage compartments of *Agc1Cre*^*ERT2*^*;Ai9*^*f/+*^ mice following tamoxifen induction at 1 month of age. Histological analyses revealed that Ai9 expression could be efficiently activated and that high abundance of Ai9 was maintained in both the AC and GP 1 month after tamoxifen delivery, suggesting sufficient Cre-mediated recombination (Supplementary Fig. 1). To specifically target Glut1 loss-of-function (LOF) in chondrocytes, we next generated *Agc1Cre*^*ERT2*^*;Glut1*^*f/f*^ (*Glut1*^*Agc1ER*^; Glut1 LOF) mice. Conditional deletion of *Glut1* started at 1 month of age, when longitudinal bone growth continues to occur as GP chondrocytes proliferate and progress through a hypertrophic process. In contrast, AC is fully developed and stable at this age. Successful removal of Glut1 was histologically verified in both cartilage compartments in Glut1 LOF mice (Fig. 2a, b).

**Fig. 3:**
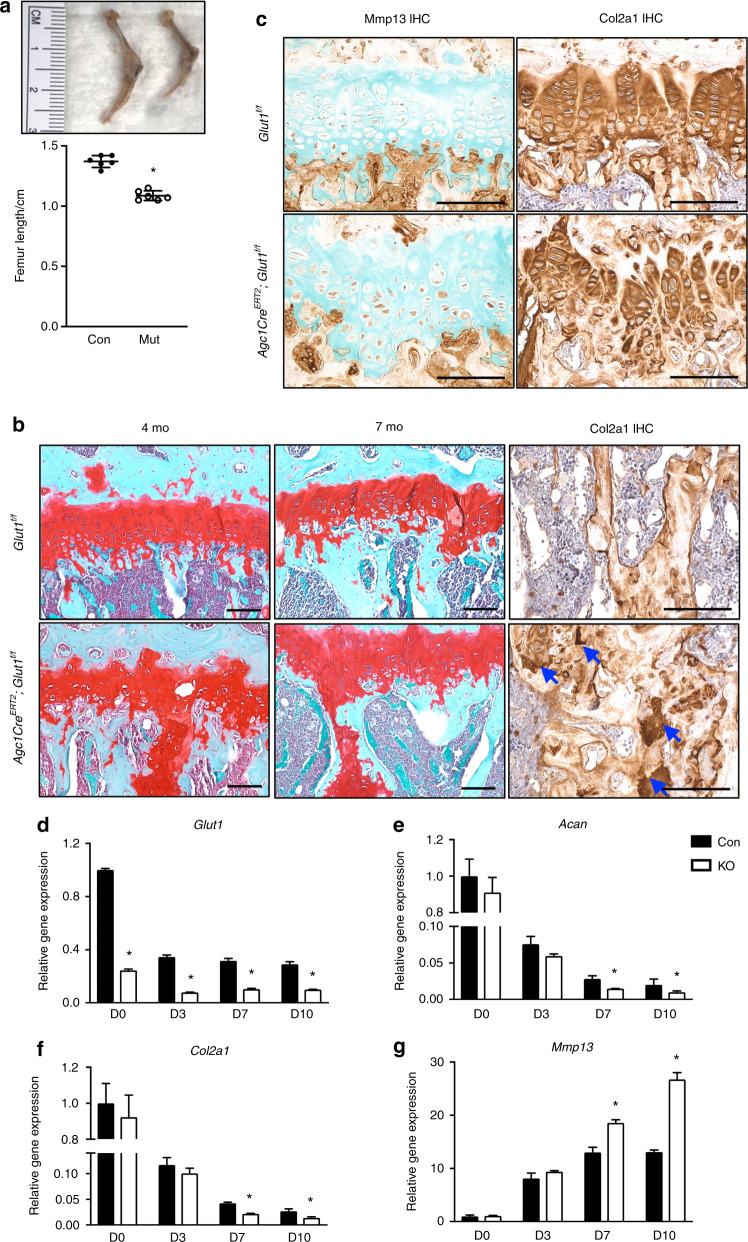
Deletion of Glut1 in chondrocytes leads to shortened and disorganized GPs, reduced matrix deposition, and skeletal dysplasia. **a** Whole hind limbs and femur lengths of control and mutant Glut1 LOF mice at 4 months. The data are the mean ± SD. *N* = 6. *P < 0.05. **b** Safranin O/Fast Green staining and immunostaining for Col2a1 in tibial sections of control and Glut1 LOF mice at 4 months and 7 months. The blue arrows denote the Col2a1-positive cartilage remnants in the metaphyses of Glut1 LOF mice. *N* = 6. Scale bar, 100 μm. **c** Immunostaining for Mmp13 and Col2a1 in the growth plates of tibia sections of control and Glut1 LOF mice at 4 months. *N* = 6. Scale bar, 100 μm**. d** RT-qPCR analyses of *Glut1*, *Col2a1*, *Acan*, and *Mmp13* in control and Glut1 KO primary GP chondrocytes at the indicated time points over the course of hypertrophic differentiation. All mRNA abundances were normalized to the abundance of *β-actin* and then normalized to the abundances in the controls at day 0. *N* = 3. *P < 0.05 relative to the controls

By 4 months, the Glut1 LOF mice exhibited shortening of long bones with no other overt abnormalities (Fig. 3a). Histological characterization of the mutant hind limbs demonstrated a shortened and disorganized GP with an overall decrease in Col2a1 in mutant GPs (Fig. 3c and Supplementary Fig. 2a). Notably, we observed increased amounts of cartilage remnants within bony trabeculae in the metaphysis, as indicated by increased amounts of Col2a1-positive and proteoglycan-rich matrix underneath the GP (Fig. 3b). In severe cases, a second horizontal cartilage plate distal to the original GP was observed crossing over the diaphysis in the mutants (data not shown). The cartilage remnants persisted through adulthood with no further resorption through the last time point at 14 months (Fig. 3b and Supplementary Fig. 3). Unexpectedly, increased levels of Mmp13, a marker of chondrocyte terminal hypertrophy, were observed in Glut1 LOF GPs (Fig. 3c), suggesting that the incomplete resorption of the cartilage matrix was not due to delayed differentiation. In addition, comparable osteoclast activity was observed in Glut1 LOF mice, as reflected by Oc.S/BS and N.Oc/BS values in the metaphysis region that were similar to those of the control mice (Supplementary Fig. 4), indicating that the persistent cartilage remnants in Glut1 LOF mice were not due to reduced osteoclast resorption but rather were likely secondary to a qualitative change in matrix properties. We also performed micro-CT scans to examine the trabecular bone quality in the metaphysis region and found no significant difference in BV/TV between Glut1 LOF mice and their littermate controls at 4 months and 7 months of age (Supplementary Fig. 5), indicating that the cartilage remnants induced by loss of Glut1 in chondrocytes do not alter bone quality.

To confirm these in vivo findings, we isolated primary GP chondrocytes and performed longitudinal differentiation assays following elimination of Glut1. Glut1 LOF cells expressed significantly lower levels of the cartilage matrix genes *Acan* and *Col2a1* than the control cells, consistent with the decreased matrix synthesis seen in Glut1 LOF GPs (Fig. 3d–f). Consistent with the elevated Mmp13 staining in Glut1 LOF GPs, *Mmp13* expression was markedly enhanced in Glut1 LOF cells as they progressed through an in vitro maturation process (Fig. 3g), reinforcing the notion that the increased amounts of cartilage remnants in the mutant metaphyseal bone were not simply caused by delayed hypertrophy or lack of MMP13 expression. In fact, Col10a1 was normally expressed in the hypertrophic zone of Glut1 LOF GPs and showed no obvious difference relative to the control animals (Supplementary Fig. 2b). These findings together suggest that Glut1 and glucose metabolism are critical for GP elongation and cartilage matrix synthesis and turnover/remodeling.

### Glut1 is indispensable for the maintenance of AC homeostasis

**Fig. 4:**
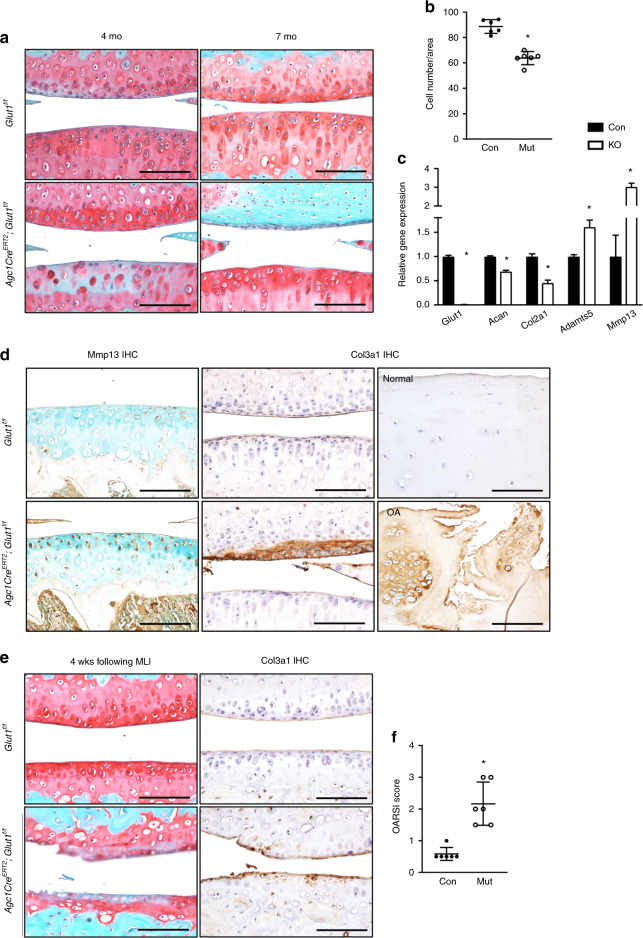
Deletion of Glut1 in chondrocytes decreases cellularity, disrupts cellular homeostasis, and ultimately causes cartilage fibrosis in AC. **a** Safranin O/Fast Green staining of knee sections of control and Glut1 LOF mice at 4 months and 7 months. *N* = 6. Scale bar, 100 μm. **b** Histomorphometric analyses of chondrocyte numbers in knee sections at 4 months. The data are the mean ± SD. *N* = 6. *P < 0.05. **c** RT-qPCR analyses for *Glut1*, *Col2a1*, *Acan*, Adamts5 and *Mmp13* in control and Glut1 KO primary AC chondrocytes. All mRNA abundances were normalized to that of *β-actin* and then normalized to those of the controls. The data are the mean ± SD. *N* = 3. *P < 0.05 relative to the controls. **d** Immunostaining for Mmp13 and Col3a1 in knee sections of control and Glut1 LOF mice at 4 months (*N* = 6) or of Col3a1 in biopsies of normal and osteoarthritic cartilage from human patients (*N* = 3). Scale bar, 100 μm. **e** Safranin O/Fast Green staining and Col3a1 immunostaining of knee sections of control and Glut1 LOF mice at 4 weeks following MLI injury. *N* = 6. Scale bar, 100 μm. **f** OARSI scores for the medial tibial plateau and femoral condyle at 4 weeks following MLI injury. The data are the mean ± SD. *N* = 6. *P < 0.05 compared to the controls

AC also displayed abnormalities upon loss of Glut1 postnatally. Histological analyses revealed significantly diminished cellularity in Glut1 LOF AC at 4 months (Fig. 4a), particularly in the tibia plateau, that was accompanied by reductions in proteoglycans, as indicated by decreased Safranin O staining (Fig. 4b). In addition, Glut1 ablation in primary AC chondrocytes resulted in a significant decrease in the expression of the anabolic genes *Acan* and *Col2a1* and a marked increase in the expression of the catabolic markers *Adamts5* and *Mmp13* (Fig. 4c). Mmp13 protein expression, which is typically minimal in normal AC, was substantially increased in Glut1 LOF AC (Fig. 4d, left panels). Interestingly, cartilage fibrosis developed by 7 months (Fig. 4a), as evidenced by areas of Col3a1 expression that were associated with complete loss of proteoglycans in the same region (Fig. 4d, middle). We next examined whether Col3a1, a pathological fibrosis marker, is expressed in human OA cartilage (Fig. 4d, right). In contrast to the normal cartilage obtained from patients with amputation, which lacked Col3a1, osteoarthritic cartilage from OA patients expressed a substantial amount of Col3a1 throughout the entire tissue.

We next sought to determine how Glut1 LOF can modify disease progression in an experimental OA setting. OA was introduced by MLI surgery at 3 months in control and Glut1 LOF mice in which *Glut1* was targeted for deletion at 1 month of age. As expected, control animals showed no overt lesions in their AC at 4 weeks following MLI. In contrast, severe cartilage damage was observed in Glut1 LOF AC, including severe surface fibrillations and fissures, massive loss of cartilage and diminished proteoglycan staining (Fig. 4e). Moreover, Col3a1 expression was detected at this time in Glut1 LOF mice but was nearly undetectable in controls (Fig. 4e, right). More importantly, the increased severity of OA and the exacerbation of cartilage deterioration were confirmed with the OARSI scoring system (Fig. 4f). Collectively, these data indicate that Glut1 and glucose metabolism are required for the maintenance of AC homeostasis and that AC with Glut1 LOF is less resistant to injury and more susceptible to the development of OA than normal AC.

### Glut1-mediated glucose metabolism is necessary for GP chondrocyte proliferation and AC chondrocyte survival

**Fig. 5:**
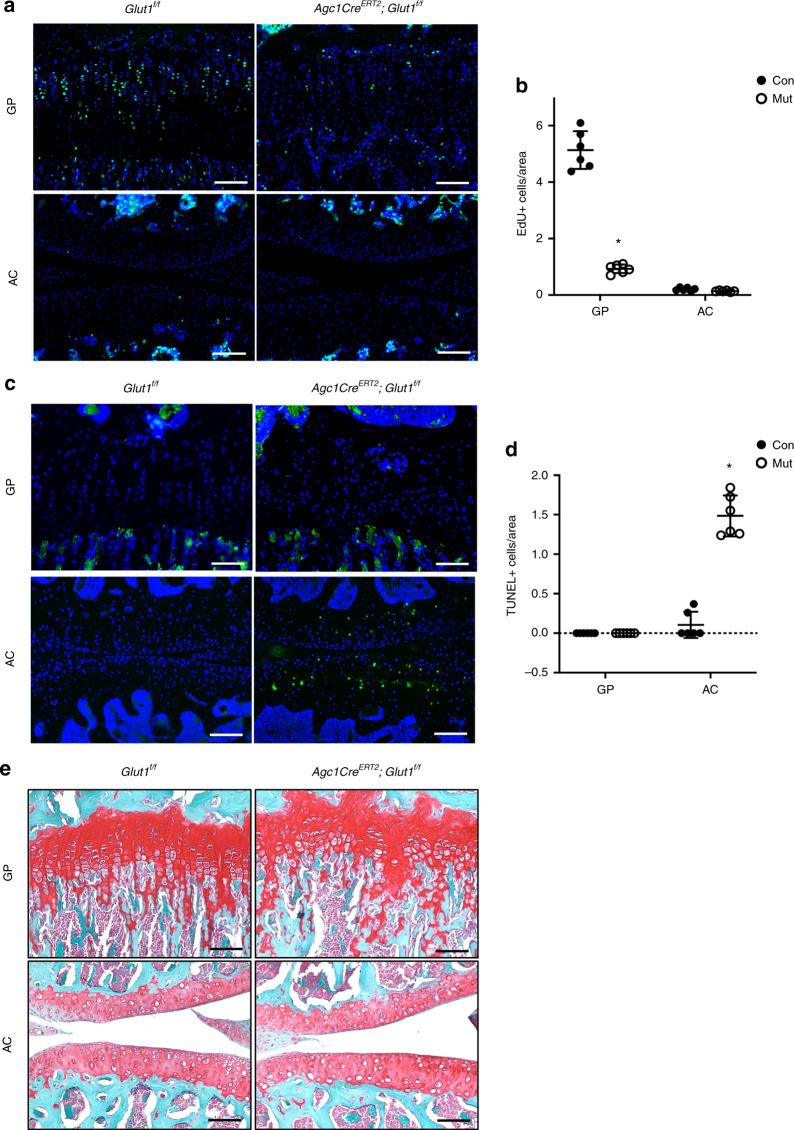
Glut1 is required for GP chondrocyte proliferation and AC chondrocyte survival. **a**–**b** EdU labeling (**a**) and quantification of EdU-positive cell counts (**b**) in the GPs and AC of control and Glut1 LOF mice at 1 week following tamoxifen induction. The data are the mean ± SD. *N* = 6. Scale bar, 100 μm. *P < 0.05 compared to the controls. **c**–**d** TUNEL staining and quantification of TUNEL-positive cell counts in the GPs and AC of control and Glut1 LOF mice 1 month following tamoxifen induction. The data are the mean ± SD. Scale bar, 100 μm. *N* = 6. *P < 0.05 compared to the controls. **e**–**f** Safranin O/Fast Green staining of tibia and knee sections of control and Glut1 LOF 1 month following tamoxifen induction. *N* = 6. Scale bar, 100 μm

We next investigated whether any cellular defects could be responsible for the shortened GP and decreased cellularity in AC seen in Glut LOF mice. Cell proliferation, measured by EdU incorporation 1 week following tamoxifen induction, was robust in the control GPs, especially in the columnar region, as indicated by quantification of EdU labeling. In contrast, Glut1 deletion caused a remarkable reduction in cell proliferation to a nearly undetectable level (Fig. 5a upper panel, b). Unlike in GP chondrocytes, EdU incorporation was barely visible in either group of AC chondrocytes, likely due to the lack of involvement of AC chondrocytes in limb growth (Fig. 5a lower panel, b). In contrast, GP TUNEL staining demonstrated no apparent apoptosis in either the control or Glut1 LOF GPs at 2 months (Fig. 5c upper panel, d). However, AC with Glut1 deletion had extensive apoptosis, while the control AC showed virtually no apoptosis (Fig. 5c, lower, d). Notably, this early apoptosis was transient and no longer detected at later stages (data not shown). Thus, Glut1 is critical for the normal proliferation of GP chondrocytes but dispensable for their survival; however, Glut1 is necessary for the viability of AC chondrocytes in fully mature AC. Indeed, Glut1 LOF mice exhibited shortened and disorganized GPs and decreased cellularity in the AC at later times (Figs. 3b, 4a, 5e).

### Glutamine oxidation is enhanced as an alternative metabolic pathway in Glut1 LOF chondrocytes

**Fig. 6:**
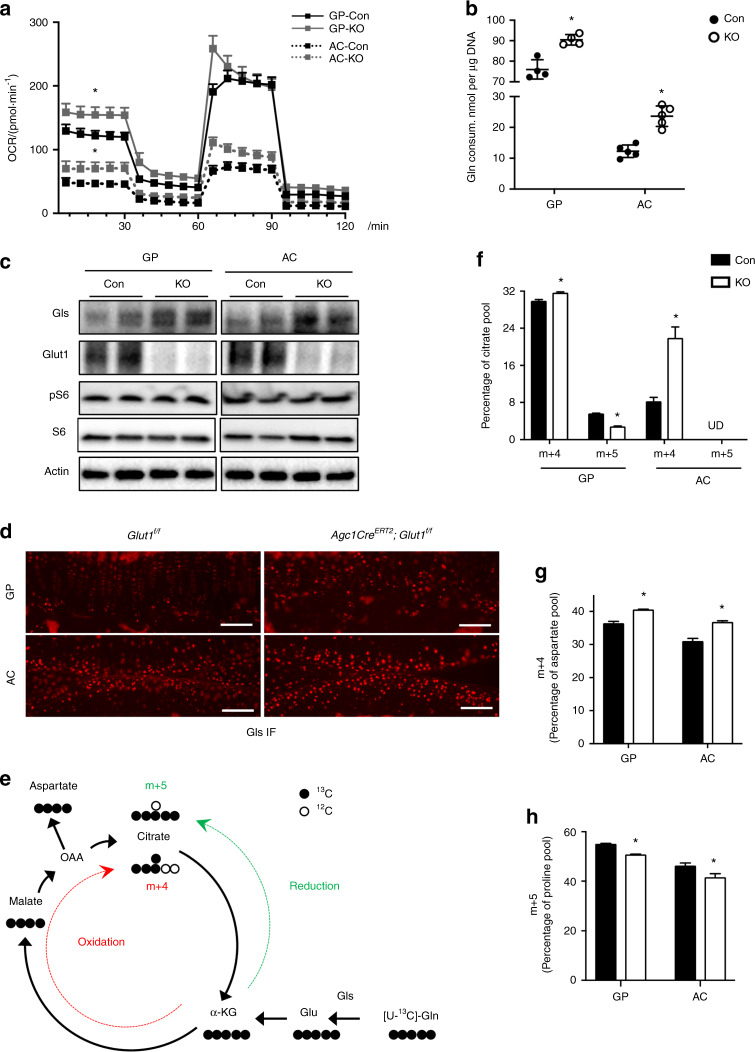
Both GP and AC chondrocytes exhibit enhanced glutamine metabolism in the absence of glucose availability. **a** A Seahorse assay was used to assess the oxygen consumption rates (OCRs) of control and Glut KO primary GP and AC chondrocytes. The data are the mean ± SD. *N* = 8. *P < 0.05 relative to the controls. **b** Glutamine consumption by control and Glut1 KO primary GP and AC chondrocytes for 24 h. The data were normalized to the genomic DNA content and are expressed as the mean ± SD. *N* = 5. *P < 0.05. **c** Western blot analyses of glutaminase (Gls), Glut1, pS6 and S6 protein levels in control and Glut KO primary GP and AC chondrocytes. *N* = 3. **d** Immunofluorescence staining for Gls in tibia and knee sections of control and Glut1 LOF mice at 2 months. *N* = 6. Scale bar, 100 μm. **e** Graphical depiction of glutamine metabolism tracing with [U-^13^C] glutamine. The black filled circles denote ^13^C, and the black open circles indicate ^12^C. Gln glutamine, Glu glutamate, α-KG α-ketoglutarate, OAA oxaloacetate. Glutamine is converted to citrate through glutamine oxidation, as indicated by the red dashed line. The glutaminolysis pathway contains glutamine oxidation in the TCA cycle and the malate-aspartate shuttle. Glutamine can also be converted via reductive carboxylation, as denoted by the green dashed line. **f**–**h** Fractions of citrate m + 4 and m + 5 (**f**), aspartate m + 4 (**g**), and proline m + 5 (**h**) after culture of control and Glut KO GP and AC chondrocytes for 24 h with [U-^13^C] glutamine. The data are the mean ± SD. *N* = 3. *P < 0.05 compared to the controls

As glycolysis is the most important energy-producing pathway in chondrocytes, we anticipated that metabolic alterations due to reduced glucose uptake would occur in association with phenotypic changes in Glut1 LOF mice. Interestingly, Seahorse analysis of the mitochondrial oxygen consumption rate (OCR) demonstrated an increase in the OCR in Glut1 LOF GP and AC chondrocytes compared to controls with normal Glut1 expression (Fig. 6a). Notably, GP chondrocytes have a much higher rate of oxidative phosphorylation than AC chondrocytes. Glut1-deficient AC chondrocytes had relatively greater increases in oxygen consumption than GP chondrocytes, in part due to their lower basal levels of oxidative phosphorylation (Fig. 6a). As expected, the extracellular acidification rate (ECAR), an indicator of glycolysis, was substantially reduced in both types of Glut1 LOF chondrocytes (Supplementary Fig. 6a). The elevated mitochondrial respiration in Glut1 LOF cells led to slightly increased ATP production with no alteration of basal reactive oxygen species (ROS) generation (Supplementary Fig. 6b, c), implicating induction of alternative metabolic pathways such as glutamine metabolism. Glutaminolysis fuels the TCA cycle by catabolizing glutamine to α-ketoglutarate (α-KG). Indeed, glutamine consumption during the 24 h period was significantly increased in both GP and AC Glut1 LOF chondrocytes (Fig. 6b). It is important to note that in the absence of Glut1, AC chondrocytes exhibited a much greater increase (>2-fold) in glutamine utilization than GP chondrocytes (Fig. 6b), possibly because the baseline glutamine consumption was already at a much higher level in control GP chondrocytes. These differences in glutamine utilization partially explain the discrepancy in basal OCR between the two types of chondrocytes. Consistent with the enhanced glutamine flux, the levels of glutaminase (Gls), the primary enzyme initiating glutamine catabolism, were markedly increased in Glut1 LOF chondrocytes of both GPs and AC in vitro (Fig. 6c) and in vivo (Fig. 6d). Since Glut1-mediated glucose metabolism has been shown to positively regulate protein synthesis in osteoblasts,^15^ we performed western blotting to determine whether the expression of phosphorylated s6 kinase, a key enzyme involved in protein synthesis, was altered in Glut1 LOF chondrocytes. Unlike in osteoblasts, Glut1 deficiency did not alter phosphorylated s6 kinase expression in either GP or AC chondrocytes (Fig. 6c).

Thus, these findings suggested that glucose deprivation in chondrocytes results in enhanced glutaminolysis, which serves as an alternative metabolic pathway and compensates for deficits in energy and biosynthetic precursors to maintain cell survival and tissue homeostasis. To verify this finding, we used uniformly ^13^C-labeled glutamine to trace the downstream metabolic fluxes. Briefly, following adenoviral infection and recovery, *Glut1*^*f/f*^ GP and AC chondrocytes were incubated with U-^13^C glutamine for 24 h, and the contribution of the tracer to downstream metabolites was determined by measuring the isotopolog pattern (Fig. 6e). As expected, U-^13^C glutamine was incorporated into the TCA cycle, and the citrate m + 4 isotopolog (Fig. 6f), as well as the aspartate m + 4 isotopolog, as part of the malate-aspartate shuttle (Fig. 6g), were enriched in both GP and AC Glut1 LOF chondrocytes. Glutamine-derived α-KG (α-ketoglutarate) can be utilized to generate citrate in the TCA cycle. Although the oxidation pathway is preferred in many cancer cells, glutamine can also be converted to citrate via reductive carboxylation during conditions of hypoxia or mitochondrial dysfunction.^27,28^ We then further evaluated glutamine utilization by comparing the citrate m + 4 isotopolog and m + 5 isotopolog. First, glutamine was more readily metabolized to citrate in GP chondrocytes, as reflected by the higher enrichment of citrate m + 4 and m + 5 isotopolog (35.6% altogether) compared to that in AC chondrocytes (9.1% in total) (Fig. 6f). Second, relative to reductive carboxylation (citrate m + 5 isotopolog), glutamine incorporation into citrate occurred primarily through oxidation in the TCA cycle (citrate m + 4 isotopolog) in normal GP chondrocytes, while there was no evidence of reductive carboxylation of glutamine in either control or Glut1 LOF AC chondrocytes (Fig. 6f). However, in Glut1 GP LOF cells, reductive carboxylation of glutamine metabolism was suppressed, whereas oxidation was increased (Fig. 6f), indicating that energy production was prioritized under circumstances of glucose limitation.

Conversely, the contribution of glutamine to synthesis of other amino acids, such as proline, was markedly reduced in chondrocytes. Therefore, there was enhanced glutamine flux due to glucose deficiency, but it was reprogrammed toward increased glutamine oxidation, an energy-producing pathway, rather than amino acid biosynthesis, as exemplified by the reduced production of proline from glutamine.

### Enhanced glutamine-dependent α-KG production results in altered collagen processing in Glut1 LOF cartilage

**Fig. 7:**
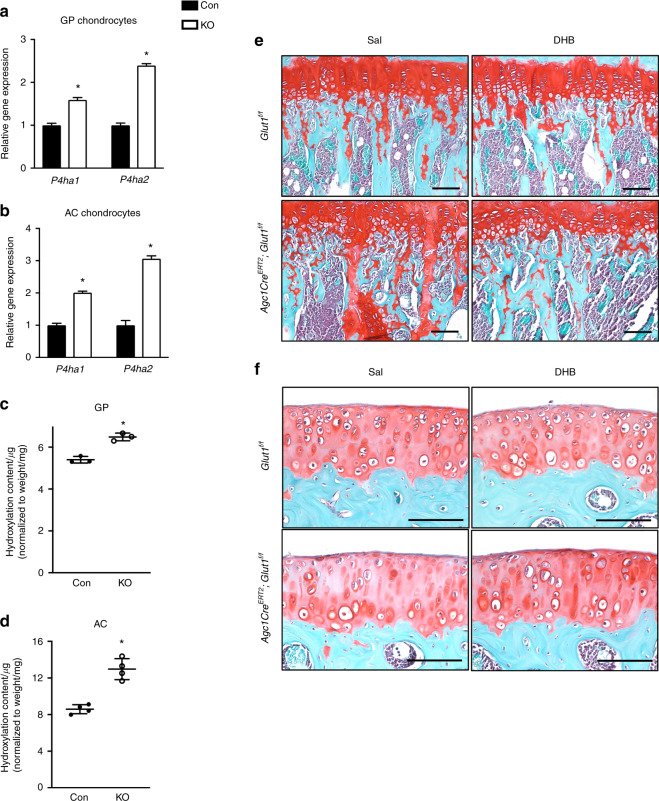
Inhibition of P4h in vivo normalizes increased the amount of cartilage remnants in the metaphyseal region in Glut1 LOF mice. **a**–**b** RT-qPCR analyses for *P4ha1* and *P4ha2* in control and Glut1 KO primary GP chondrocytes. The data are the mean ± SD. *N* = 3. *P < 0.05 relative to the controls. **c**–**d** Hydroxyproline levels in control and Glut1 LOF neonatal growth plates and 3-week-old articular cartilage from femoral heads. All data are normalized to the tissue weight. The data are the mean ± SD. *N* = 3 or 4 biologically independent samples. *P < 0.05 compared to the controls. **e**–**f** Safranin O/Fast Green staining of tibia and knee sections of 3-month-old control and Glut1 LOF mice following DHB or saline treatment for 2 months. *N* = 5. Scale bar, 100 μm

Previous studies have shown that enhanced glutamine metabolism to α-KG in chondrocytes increases collagen hydroxylation in the cartilage matrix, leading to abundant cartilage remnants in bony trabeculae.^29^ To investigate whether the presence of cartilage remnants in the metaphyseal bones of Glut1 LOF mice could be similarly a result of metabolically induced collagen modification, we first assessed genes associated with collagen-modifying enzymes. Our results showed that collagen prolyl-4-hydroxylases (P4hs), including both isoforms previously identified in chondrocytes, *P4ha1* and *P4ha2,*^30^ were upregulated in both Glut1 LOF GP and AC chondrocytes (Fig. 7a, b). Notably, the main enzyme isoform, *P4ha2*, showed the greatest induction. Proline hydroxylation is known to increase the stability of collagen triple helices.^31^ Given the increased enzyme levels and availability of the metabolic cosubstrate α-KG, we anticipated alterations in collagen processing. Indeed, the hydroxyproline content in cartilage collagen was increased in both Glut1 LOF GP and AC explants (Fig. 7c, d). Such collagen modifications increase the resistance of the cartilage matrix to protease-mediated breakdown.^32^ Thus, it is plausible that the MMP13 levels in Glut1 LOF cartilage are increased in an attempt to degrade the modified cartilage matrix (Figs. 3c, 4d). Finally, to validate the importance of P4h for cartilage remnants in Glut1 LOF mice, 1-month-old control and Glut1 LOF mice were treated with DHB every other day for 2 months. DHB is a selective P4h inhibitor^33^ and has been safely used in mice to inhibit P4h-mediated collagen processing and deposition.^34^ Histological examination revealed that in vivo blockade of P4h with DHB remarkably normalized the increased amount of cartilage remnants seen in the metaphyseal region in Glut1 LOF mice (Fig. 7e). In contrast, this P4h inhibitor had no effect on the shortening of GPs or on the decreased cellularity in AC (Fig. 7e, f). Thus, our results show that in addition to enhanced glutamine-derived oxidative phosphorylation, Glut1 LOF chondrocytes also have increased prolyl hydroxylase activity. Since collagen P4h is an α-KG-dependent enzyme that catalyzes proline hydroxylation to promote the formation of collagen triple helices, glutamine-derived α-KG is likely a key metabolite responsible for the increased oxidative phosphorylation and collagen overmodification in Glut1 LOF mice.

## Discussion

The present work provides genetic and metabolic evidence that Glut1-mediated glucose metabolism is critical for postnatal GP growth and maintenance of AC homeostasis. Deletion of Glut1 in chondrocytes during early postnatal development disrupts GP function and results in reduced longitudinal bone growth by interfering with chondrocyte proliferation and matrix synthesis and processing. Inhibiting Glut1 in AC, on the other hand, results in diminished cellularity and in imbalanced tissue anabolism and catabolism, with ultimate progression to cartilage fibrosis. Moreover, Glut1 LOF AC has increased susceptibility to injury-induced OA. Despite the disparate cellular phenotypes caused by Glut1 LOF, our study provides the first evidence of metabolic plasticity exhibited by both GP and AC chondrocytes that allows these cells to switch to glutamine oxidation to support energy production when glucose metabolism is compromised. Collectively, our work highlights the pivotal and distinct roles of Glut1-mediated glucose metabolism in rapidly proliferative GP chondrocytes and metabolically inactive AC chondrocytes.

Recent studies have uncovered the pivotal role of Glut1 in embryonic cartilage development. In one study, ablation of Glut1 in osteoprogenitor cells led to a disorganized and prolonged zone of hypertrophic chondrocytes until E18.4, leading to impaired bone mineralization and formation via suppression of Runx2 expression.^15^ Similarly, in another study, genetic deletion of Glut1 in mesenchymal cells led to impairment of limb development, primarily due to decreased chondrocyte proliferation and hypertrophy since cell survival was largely unaffected by loss of Glut1.^16^ The findings of our present postnatal studies are consistent with recent findings in embryos. Postnatal GP chondrocytes, particularly those residing in the flat columnar zone, are highly proliferative and primarily utilize glucose through glycolytic processes to not only accelerate the output of ATP but also allow diversion of glycolytic intermediates for biomass synthesis in support of rapid cellular proliferation.^35^ Despite its inefficient ATP production, glycolysis is the most important energy-producing pathway in GP chondrocytes.^29^ Thus, to survive glucose deprivation, GP chondrocytes decrease activities that require a steady supply of both energy and metabolites for use as building blocks;^36^ these adaptations are observed in Glut1-deficient GP chondrocytes. First, proliferation is dramatically arrested in the columnar zone in Glut1 LOF GPs. Second, glucose limitation affects matrix production, as reflected by decreased deposition of collagen and proteoglycans in Glut1 LOF GPs as well as reduced expression of cartilage matrix genes in vitro. These acquired adaptations by GP chondrocytes at least partly circumvent cell death in the absence of glucose uptake. Future studies are warranted to understand the biochemical basis for glucose metabolism to control the proliferation and biosynthetic activity of GP chondrocytes. In contrast to the decreased chondrocyte hypertrophy shown in embryonic GP chondrocytes with Glut1 LOF,^16^ the same process was largely unaffected by Glut1 LOF in the early postnatal GP, suggesting that there are differential requirements of glucose metabolism in embryonic and postnatal chondrocyte hypertrophy. In addition, our findings could have clinical implications. The predilection of proliferating GP chondrocytes for glucose described here provides a plausible explanation for the poor longitudinal growth experienced by some children and adolescents who are fed a chronically low-carbohydrate diet.^37^ In this regard, our findings argue that the main factors regulating the lengthening of the postnatal skeleton by GPs are chondrocyte proliferation and appropriate matrix remodeling/turnover but not chondrocyte hypertrophy, as previously suggested.^38,39^

Unlike GPs, where chondrocytes undergo a progressive maturation process called chondrogenesis followed by endochondral ossification, AC exhibits a unique feature: AC chondrocytes maintain a consistent phenotype over time. While extensively studied, the manner in which transient (GP) and permanent (AC) chondrocytes differ and how they acquire and develop divergent developmental and functional paths during postnatal life remain unclear.^40^ Cellular energy metabolism is increasingly being recognized as an important driver of cellular phenotype. Here, we uncovered important disparities in glucose metabolism between GP and AC chondrocytes. The high demand for glucose uptake by AC chondrocytes explicitly explains the requirement of glucose for cell survival and the increased apoptosis observed in Glut1 LOF AC. In contrast, glucose metabolism is dispensable for GP chondrocyte survival. Second, although both types of chondrocytes favor glycolysis for generating energy and biomass under normal conditions,^22,29,41^ AC chondrocytes utilized mitochondrial respiration much less than GP chondrocytes, as indicated by their smaller basal OCR (46.84 ± 0.89 vs 123.22 ± 0.89 pmol·min^−1^ per 5 × 10^4^ cells). Furthermore, comparison of the ECAR/OCR ratio, an indicator of glycolysis dependence relative to oxidative phosphorylation, indicated that AC chondrocytes (0.67 ± 0.02) seemed to be more dependent on glycolysis than GP chondrocytes (0.38 ± 0.02) regardless of aerobic conditions. The higher rates of glycolysis present in AC chondrocytes may be necessary to provide the substrates required for the anabolic activities occurring in AC chondrocytes, since the glycosaminoglycan side chains of the AC proteoglycan-rich matrix are actively synthesized and degraded at a higher rate than the turnover of the protein cores, which are metabolized at a much lower rate.^42^ OA cartilage is characterized by decreased expression of several glycolytic enzymes and an increase in mitochondrial respiration under basal conditions.^43,44^ Recently, metabolic reprogramming in AC chondrocytes has been linked to cell hypertrophy and associated catabolic mechanisms, most notably those related to phenotypes acquired by OA cartilage.^22^ This finding is supported by our results indicating that loss of Glut1 in AC chondrocytes causes increased mitochondrial respiration, ultimately leading to an imbalance toward catabolic pathways in AC, as highlighted by enhanced Mmp13 expression and significant loss of proteoglycan content in some regions. Future studies will be necessary to elucidate the precise mechanisms by which metabolic reprogramming contributes to the pathogenesis of OA. Moreover, it is important to note that Glut1 LOF AC is able to overcome transient cell death and maintain cell survival by upregulating mitochondrial respiration under glucose stress, suggesting that AC chondrocytes exhibit metabolic flexibility similar to that of GP chondrocytes; however, this metabolic switch is unable to compensate for the loss of glucose metabolism in AC under extreme instances, such as injury.

Here, we provide evidence that primary chondrocytes from GPs and AC display metabolic plasticity under stress conditions due to reduced glucose uptake as a result of Glut1 LOF. Metabolic plasticity is presumably important for chondrocytes to be able to best utilize available nutrients under unfavorable conditions, thus meeting diverse cellular demands associated with proliferation, differentiation, and matrix synthesis and secretion. For most mammalian cells, the metabolic pathways required for cell survival and growth are predominantly fueled by two of the most abundant nutrients, glucose and glutamine.^45^ Here, we demonstrate that in the absence of glucose availability, both GP and AC chondrocytes increase mitochondrial respiration and maintain energy production by utilizing glutamine-derived α-KG as an alternative mitochondrial substrate. Studies have indicated that under circumstances of nutrient limitation or impaired supply of glucose-derived pyruvate to the mitochondria, maintenance of pools of TCA intermediates via glutamine oxidation is essential for the survival of some rapidly proliferating cells.^46,47^ This is consistent with our observations from GP chondrocytes showing that cell viability was maintained via promotion of glutamine oxidation with concomitant suppression of glutamine reductive carboxylation. We and others have demonstrated that there are remarkable reductions in cell proliferation and protein synthesis when glucose metabolism is limiting.^15,16^ Thus, engaging in glutamine oxidation enables GP chondrocytes to survive in glucose-limited conditions; however, normal proliferation and matrix production by these cells is impaired, which likely results in a need for glucose oxidation.

With regard to AC chondrocytes, it is not surprising that Glut1 LOF in AC chondrocytes results in apoptosis despite a shift to glutamine oxidation, unlike in Glut1-deficient GP chondrocytes, which do not have an increase in apoptosis. There are two possible explanations. First, AC chondrocytes are highly dependent on glucose metabolism and less versatile in metabolizing other nutrients, as exemplified by the lower glutamine consumption and fully labeled citrate levels in AC chondrocytes than in GP chondrocytes. Second, increased mitochondrial metabolism produces excess ROS, which are known to be detrimental to articular chondrocytes.^48^ Although no alterations in ROS generation were observed in Glut1 LOF AC chondrocytes, it is still possible that the normoxic culture conditions used in the current study artificially induced excess ROS production in AC chondrocytes compared to that in hypoxic conditions since AC is a naturally avascular tissue with low oxygen tension ranging from 2% to 7%.^49^ Therefore, future studies are needed to determine the regulatory mechanisms by which intrinsic glucose metabolism regulates the proliferation of GPs and the survival of AC chondrocytes.

Finally, we found that enhanced glutamine oxidation at least partially maintained the cell survival of GP chondrocytes; however, it caused unfavorable collagen modifications and consequently led to persistent cartilage remnants in the bony trabeculae. P4h is an α-KG-dependent dioxygenase that catalyzes the 4-hydroxylation of proline to promote the formation of the collagen triple helix.^50^ Both isoforms of the catalytic subunit P4ha were significantly upregulated in both types of Glut1 LOF chondrocytes. As expected, the increased enzyme levels and availability of the metabolic cosubstrate α-KG resulted in an increase in proline hydroxylation content in ex vivo-cultured Glut1 LOF cartilage. Previous studies have demonstrated that P4h-mediated collagen content alteration, specifically increased hydroxylation of proline, enhances the stability of the mature collagen triple helices deposited by chondrocytes and makes the cartilaginous matrix more resistant to protease- and osteoclast-mediated degradation and resorption in mice.^29,31^ Therefore, as expected, targeting P4h in vivo reversed the increases in cartilage remnants in the metaphyses of Glut1 LOF mice, restoring the amounts to the levels in control mice. Notably, P4h blockade showed no obvious protective effect against proteoglycan loss in AC or against the reduced cellularity that occurs in Glut1 LOF AC chondrocytes. Although collagen overmodification also occurred in Glut1 LOF AC, the manner in which this contributed to joint degeneration and may have been corrected by DHB treatment was not determined in our experiments since we only evaluated DHB-treated mice at 3 months of age.

In summary, our findings have important translational implications, as some cartilage abnormalities, such as excessive ECM remodeling and fibrosis, could be potentially associated with changes in glucose/glutamine metabolism.

## Materials and methods

### Mice

All animal studies were performed in accordance with approval of the Committees on Animal Resources in Washington University in St Louis. *Glut1*^*f/f*^ mice were generated as previously described.^51^ *Ai9* Cre reporter mice were purchased from the Jackson Laboratory.^52^ *Agc1Cre*^*ERT2*^ mice^53^ were generous gifts from Dr. Benoit de Crombrugghe (Department of Genetics, University of Texas MD Anderson Cancer Center, Houston, TX). *Agc1Cre*^*ERT2*^*;Glut1*^*f/f*^ (*Glut1*^*Agc1ER*^; Glut1 LOF) mice, *Agc1Cre*^*ERT2*^*;Rosa-Ai9*^*f/+*^ (*Rosa-Ai9*^*Agc1ER*^) mice and Cre-negative littermates (*Glut1*^*f/f*^ and *Rosa-Ai9*^*f/+*^) were viable and produced Mendelian ratios. Tamoxifen was administered daily at a dose of 1 mg per 10 g body weight for 5 consecutive days via intraperitoneal injection to 1-month-old *Glut1*^*Agc1ER*^ mice *Rosa-Ai9*^*Agc1ER*^ mice, and Cre-negative controls to remove *Glut1* alleles or induce *Ai9* expression. To determine whether Glut1 LOF modifies the disease progression of injury-induced OA, meniscal ligament injuries (MLIs) were created unilaterally in the knee joints as previously described^54^ in *Glut1*^*Agc1ER*^ mice and their littermates following tamoxifen induction at 1 month. Ethyl-3,4-dihydroxybenzoic acid (DHB; Sigma-Aldrich; #E24859) was administered intraperitoneally to 1-month-old control and Glut1 LOF mice every other day at a dose of 40 mg·kg^−1^ body weight for 2 months.

### Histological analyses

Mouse knees were collected at the indicated time points and fixed in 10% neutral buffered formalin for 3 d. These specimens were decalcified for 3 d in a formic acid decalcifier (ImmunoCal; StatLab, #1414-1), processed and embedded in paraffin, and sectioned at a thickness of 5 μm. The sections were stained with Safranin O/Fast Green to analyze phenotypic changes within the knee joint and GP. Following staining, the sections were scanned using a NanoZoomer 2.0-HT whole-slide imager (Hamamatsu), and cellularity above the tidemark in the cartilage of the tibial plateau was subsequently measured using NDP.View 2 software with the scanned images. Three sections for each specimen were examined for all quantitative histomorphometric analyses. Histological scoring of OA-like changes on the medial femoral condyle and tibial plateau was performed for *Glut1*^*Agc1ER*^ and control mice 4 weeks following MLI using the established Osteoarthritis Research Society International (OARSI) scoring system (score, 0–6).^55^ Immunohistochemical staining for Glut1 (1:200; Abcam; #ab28484), Col2A1 (1:100; Thermo Fisher Scientific; #MS235-P), Col10A1 (1:100; Quartet; #1-CO097-05), Mmp13 (1:200; Abcam; #ab39012), and Col3A1 (1:1 000; Abcam; #ab7778) was performed on paraffin sections following appropriate antigen retrieval methodologies. The signal was developed with DAB reagents (Vector Laboratories; #SK-4100), and the sections were counterstained with hematoxylin or methyl green. Immunofluorescence (IF) staining for glutaminase (Gls; 1:200, Abcam; #ab2110382) was conducted on paraffin sections following appropriate antigen retrieval, and the sections were counterstained with DAPI. The percentage of Col2A1-positive area was quantified by calculating the Col2A1-positive area over the total GP area. To evaluate osteoclast activity in the metaphysis region, TRAP staining was performed on paraffin sections. Oc.S/BS and N.Oc/BS were analyzed based on TRAP staining with a BioQuant histomorphometry system. To assess proliferation, EdU (10 μg·g^−1^ body weight) was injected daily into 1-month-old *Glut1*^*Agc1ER*^ and control mice for 3 d. Frozen sections were subjected to Click-iT EdU staining according to the manufacturer’s instructions (Invitrogen; #C10337). To assess apoptosis, TUNEL staining was performed on paraffin sections with an In Situ Cell Death Detection Kit, Fluorescein (Roche; #11684795910), according to the manufacturer’s instructions.

### Micro-CT scanning and analysis

Mouse knee joints harvested from 4- and 7-month-old mice were scanned by a Scanco VivaCT 40 scanner with an X-ray energy of 55 kVp, a current of 145 μA, an integration time of 300 ms and a voxel size of 10 μm. The bone volume fraction (BV/TV) of the bony trabeculae in the tibial metaphysis was calculated by Scanco analysis software as described previously.^56^

### Primary chondrocyte cultures

Murine costal chondrocytes and murine articular chondrocytes were isolated from the ribcages of 3-day-old wild-type (C57BL/6J) or *Glut1*^*f/f*^ pups as described previously^57^ and from the femoral heads of 3-week-old wild-type (C57BL/6J) or *Glut1*^*f/f*^ pups as described previously,^58^ respectively, with modifications. Following digestion, the chondrocytes were harvested and cultured in complete high-glucose Dulbecco’s modified Eagle’s medium (DMEM; Gibco; #31053028) supplemented with 10% fetal bovine serum (FBS; Gibco; #10437), 2 mmol·L^−1^ L-glutamine (Gibco; #A2916801), and 1% penicillin/streptomycin. According to the experimental design, the primary chondrocytes were initially seeded at a density of 50 × 10^4^ cells per well, 25 × 10^4^ cells per well, or 5 × 10^4^ cells per well in 12-well, 24-well or 96-well plates. For *Glut1*^*f/f*^ chondrocytes, the day after plating, the cells were transduced with adenoviruses expressing GFP (Ad-Con; Vector Development Lab) or Cre (Ad-Cre; Vector Development Lab) at a multiplicity of 50 in the presence of polybrene (10 μg·mL^−1^) (Millipore; #TR-1003-G) in viral infection medium (high-glucose DMEM supplemented with 2% FBS and 2 mmol·L^−1^ L-glutamine) for 24 h. Following viral infection, the cells were cultured in complete medium for 24 h to enable cell recovery and gene expression. After recovery from viral transduction, the cells were refreshed with complete medium containing high-glucose DMEM or glucose-free DMEM according to the following experimental purposes. For the longitudinal hypertrophic differentiation assay, costal chondrocytes were cultured in differentiation medium (complete medium containing 50 mg·mL^−1^ ascorbic acid and 10 mmol·L^−1^ β-glycerophosphate) and allowed to mature for up to 10 d, as indicated.

### Quantitative gene expression and western blot analyses

RNA was isolated from primary chondrocytes using an RNeasy Mini kit (Qiagen; #74134). cDNA synthesis (iScript cDNA synthesis kit; Bio-Rad; #1708841) and real-time qPCR (SYBR master mix; Bio-Rad; #172-5274) were performed according to the manufacturers’ instructions. Primers specific for *Glut1*, *Glut3*, *Glut4*, *Glut5, Glut6, Glut9, Acan*, *Col2a1*, *Mmp13*, *Adamts5*, *P4ha1*, *P4ha2*, and *β-actin* were used, and the sequences are presented in Supplemental Table 1. Western blot analyses were conducted with protein lysates from primary articular chondrocytes. The following primary antibodies were used: Gls (1:500; Abcam; #ab2110382), Glut1 (1:500; Cell Signaling Technology; #1239S), phospho-S6 protein (1:1 000; Cell Signaling Technology; #2211S), S6 protein (1:1 000; Cell Signaling Technology; #2217S) and β-actin (1:4 000; Sigma-Aldrich, #2228) antibodies.

### Metabolic assays and analyses

After treatment, aliquots of the culture media and the cell cultures were analyzed for glucose consumption, lactate production and ATP production. The extracellular glucose concentrations or glutamine concentrations were measured using a Glucose (HK) Assay Kit (Sigma-Aldrich; #GAHK-20) or a Glutamine Detection Assay Kit (Abcam; #ab197011), respectively. Glucose consumption or glutamine consumption during the period of treatment was then calculated by determining the difference in the level of each nutrient in the medium before vs after treatment. For extracellular lactate measurement, an L-Lactate Assay Kit (Eton Biosciences, Inc.; #120001100A) was used, and lactate production within the time of treatment was obtained by subtracting the lactate level in the medium before treatment from the lactate level in the medium after treatment. DNA content was evaluated with Hoechst 33342 solution (Thermo Fisher Scientific; #62249). Glucose consumption, glutamine consumption, and lactate production within 24 h were all normalized by the DNA content in each corresponding well. All aforementioned assays were performed according to the manufacturers’ instructions.

### Oxygen consumption rate measurement with a Seahorse XF Cell Mito Stress Test

Chondrocytes were plated in XF96 Seahorse plates at a density of 40 000 cells per well. Culture and treatment regimens were followed as described previously. After treatment, the cells were lifted from the regular culture plates and plated on Seahorse XF 96-well plates for 4–6 h. One hour before the test, the cells were switched to Seahorse XF base medium (Agilent Technologies; #103335-100) supplemented with 5.5 mmol·L^−1^ glucose and 2 mmol·L^−1^ GlutaMAX and further incubated in a CO_2_-free incubator for 1 h. Oligomycin, FCCP (carbonyl cyanide-p-trifluoromethoxyphenylhydrazone) and antimycin A/rotenone from a Seahorse XF Cell Mito Stress Test Kit (Agilent Technologies; #103015-100) were prepared in XF assay medium with final concentrations of 1 mmol·L^−1^, 0.5 mmol·L^−1^ and 1 mmol·L^−1^, respectively, and were serially injected to measure the OCRs of cells in an XF96 plate. ATP production and ROS generation were calculated based on the Seahorse data according to the manufacturer’s instructions.

### Hydroxyproline content measurement with cartilage explant cultures

GP cartilage at P5 or AC cartilage at P14 was carefully dissected from metaphyseal regions or femoral heads of *Glut1*^*Agc1ER*^ and *Glut1*^*f/f*^ pups, respectively. The explants were cultured in 24-well plates and treated with 4-hydroxytamoxifen (Sigma-Aldrich; #H7904) at a concentration of 10 μmol·L^−1^ for 72 h. Following treatment, the explants were switched to regular complete medium and cultured for 10 d. Upon completion of culture, the explants were removed from plates and examined for hydroxyproline content using a Hydroxyproline Assay Kit (Abcam; #ab222941). The hydroxyproline content was normalized to the tissue weight.

### Glucose and glutamine labeling experiments and intracellular metabolite analyses

*Glut1*^*f/f*^ articular chondrocytes and costal chondrocytes were isolated and seeded at the desired densities as described above. Following adenoviral transduction and recovery, 2 mmol·L^−1^ [U-^13^C_5_] glutamine (Cambridge Isotope Laboratories; #CLM-1822) was added to glutamine-free complete medium. After 24 h of labeling, the cells were harvested and extracted as previously described.^59^ The samples were analyzed with a Luna aminopropyl column (3 μmol·L^−1^, 150 mm × 1.0 mm ID, Phenomenex) coupled to a Dionex UltiMate^®^ 3000 RSLCnano LC system. The column was used in hydrophilic interaction (HILIC) mode with the following mobile phases and gradient: A = 95% water, 5% acetonitrile (ACN), 10 mmol·L^−1^ ammonium hydroxide (NH_4_OH), 10 mmol·L^−1^ ammonium acetate (NH_4_Ac); B = 95% ACN, 5% water; 100%–0% B from 0–45 min and 0% B from 45–50 min. The flow rate was 50 μL·min^−1^. MS detection was carried out on a Thermo Q Exactive Plus mass spectrometer in negative mode at 70 000 resolving power.

### Statistical analyses

All data are expressed as the mean ± SD. The results were analyzed with GraphPad Prism (GraphPad Software Inc.). Comparisons between two groups were performed using two-tailed unpaired Student’s *t* tests. One-way analysis of variance (ANOVA) was used when comparing multiple groups and was followed by the Bonferroni test as appropriate for subsequent pairwise (group) comparisons. A *P* value < 0.05 was considered to indicate statistical significance.

## Supplementary information

Suppl Fig. 1

Suppl Fig. 2

Suppl Fig. 3

Suppl Fig. 4

Suppl Fig. 5

Suppl Fig. 6

Supplementary information
