# Supplementary figures and images for: Deletion of Glut1 in early postnatal cartilage reprograms chondrocytes toward enhanced glutamine oxidation
Source: Bone Res. 2021 Aug 23;9:38. doi: 10.1038/s41413-021-00153-1 (PMC8382841; doi:10.1038/s41413-021-00153-1)

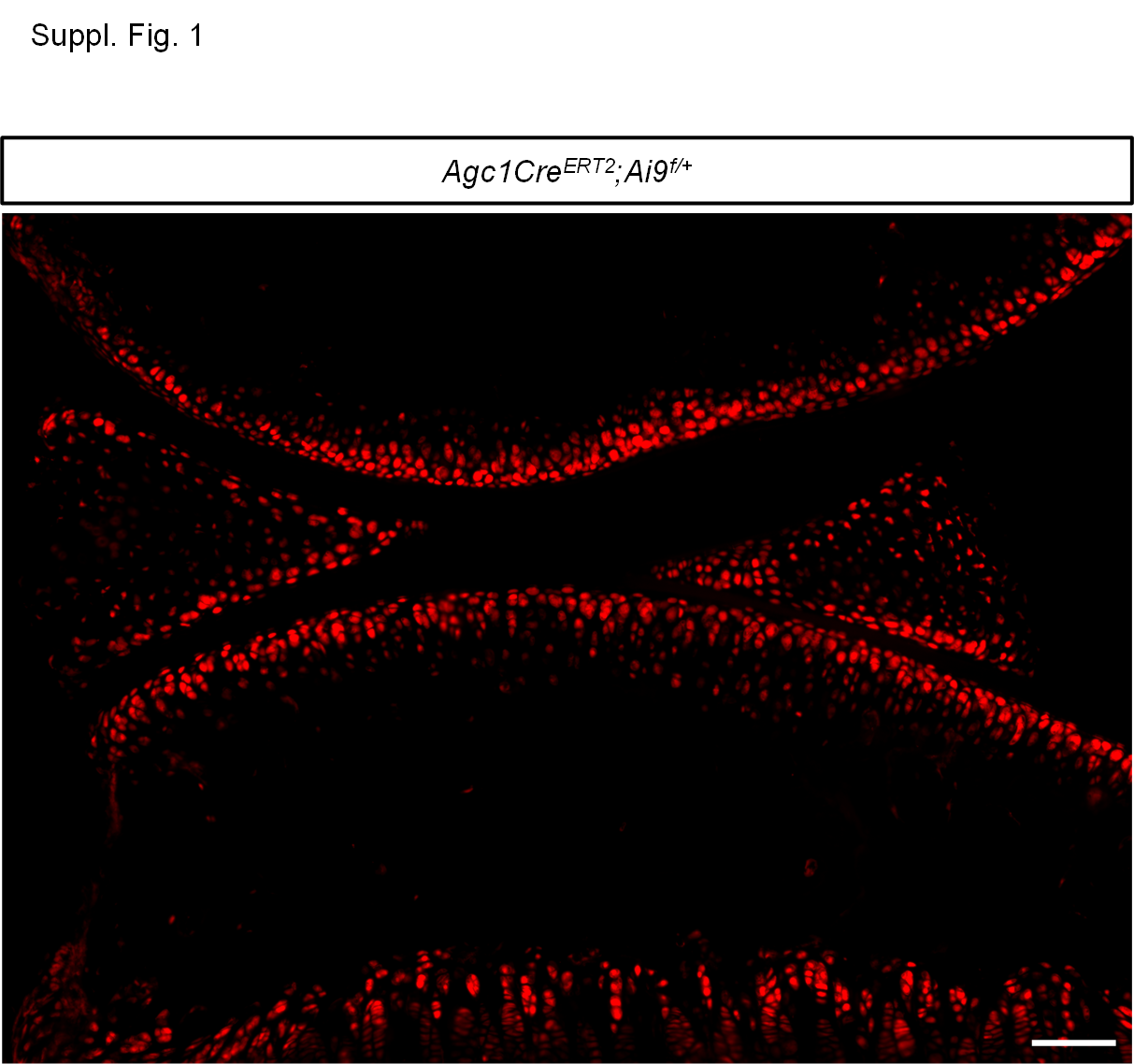

Supplement: Supplementary file 1 — Suppl Fig. 1 [file 41413_2021_153_MOESM1_ESM.tif]

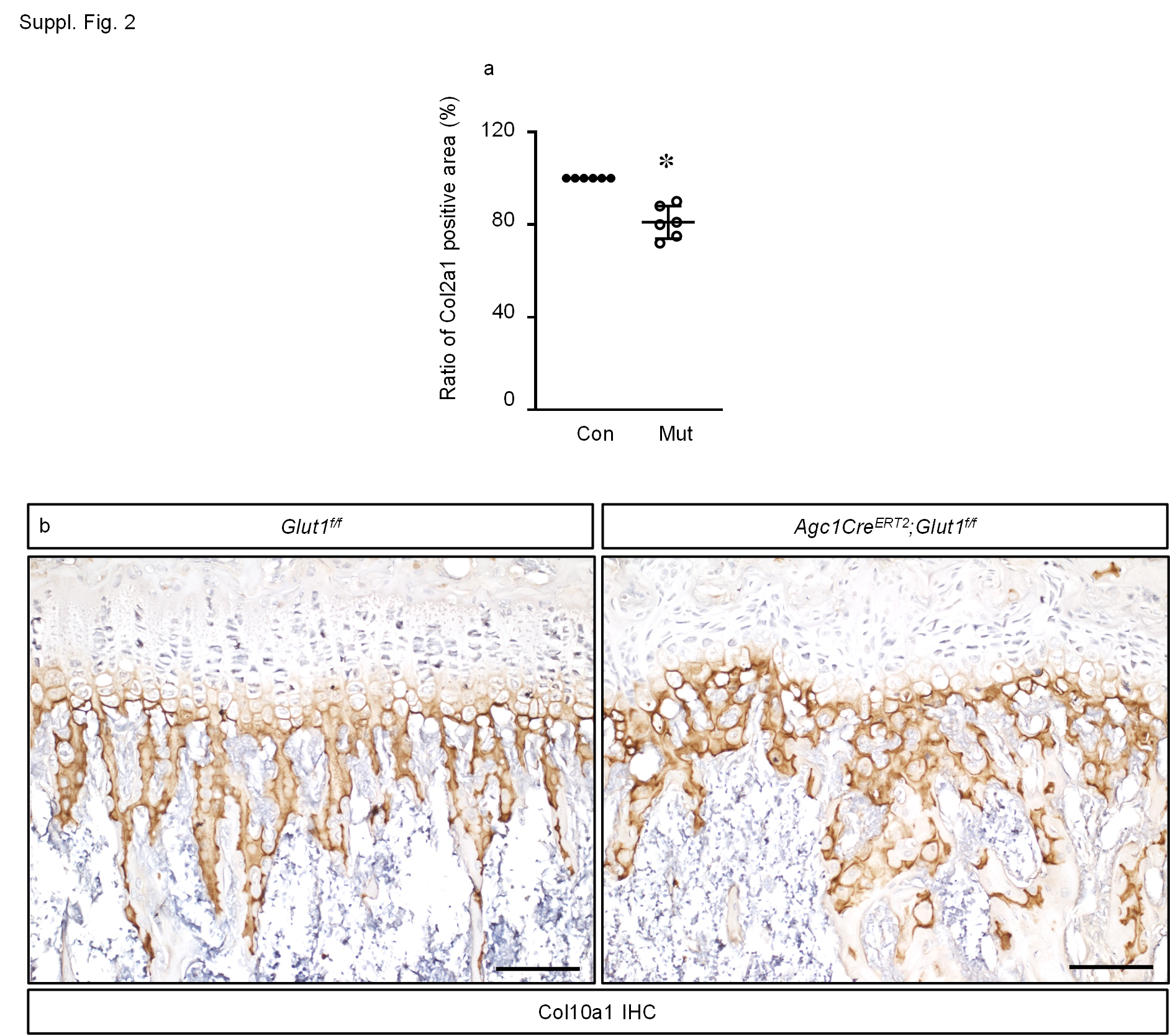

Supplement: Supplementary file 2 — Suppl Fig. 2 [file 41413_2021_153_MOESM2_ESM.tif]

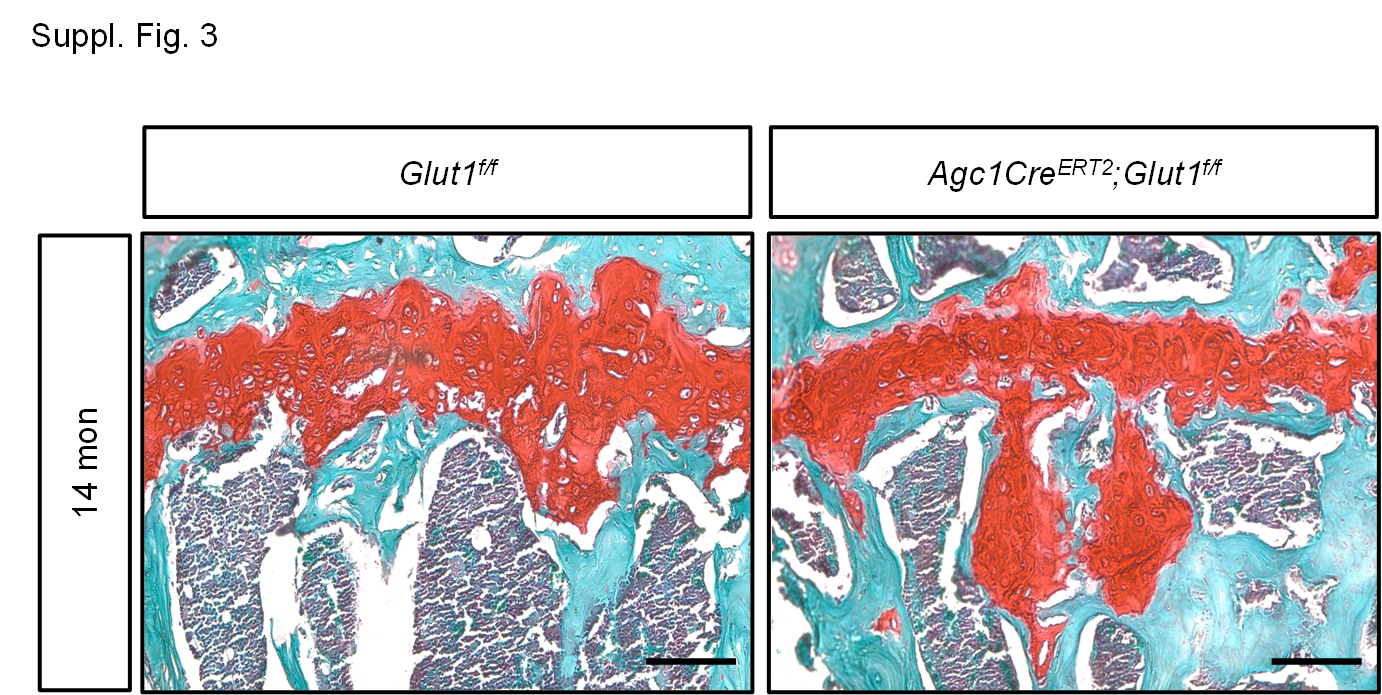

Supplement: Supplementary file 3 — Suppl Fig. 3 [file 41413_2021_153_MOESM3_ESM.tif]

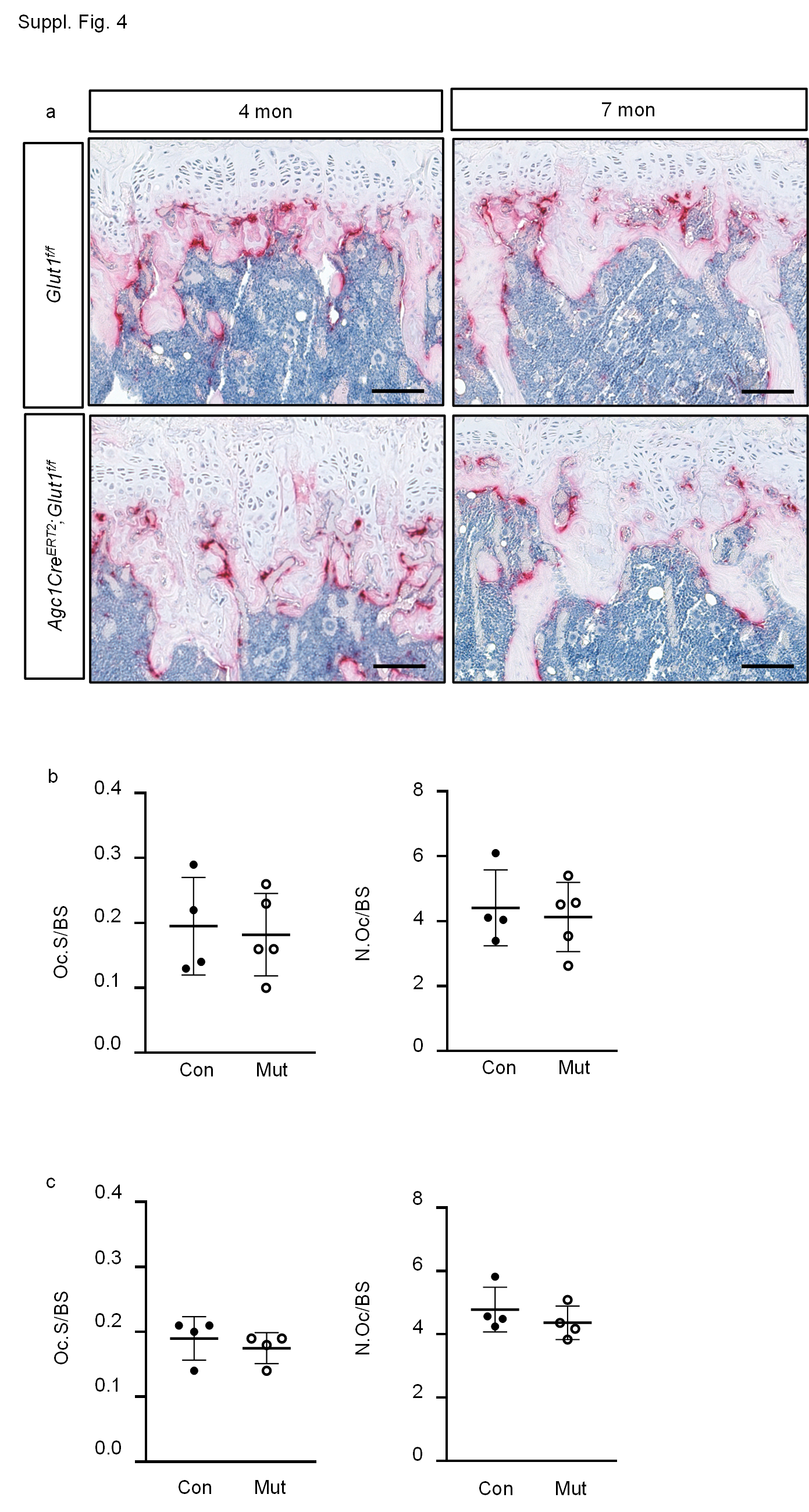

Supplement: Supplementary file 4 — Suppl Fig. 4 [file 41413_2021_153_MOESM4_ESM.tif]

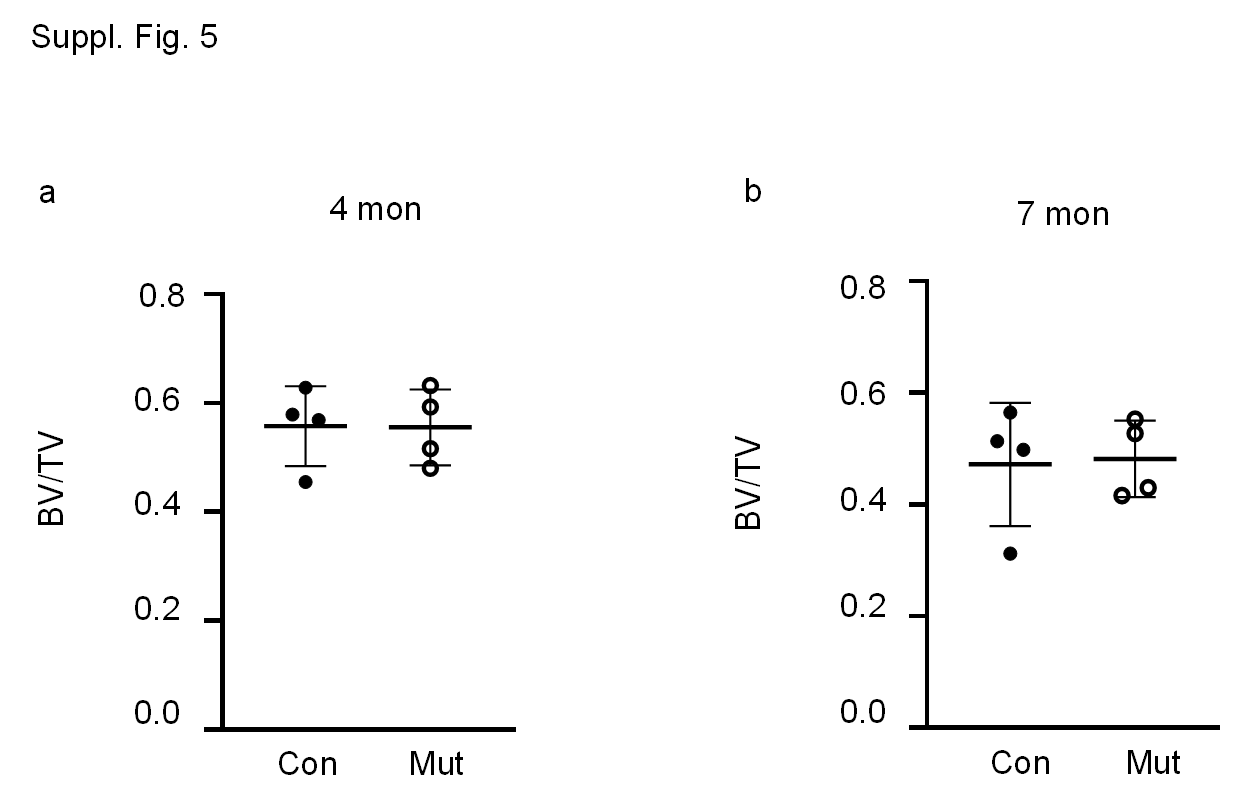

Supplement: Supplementary file 5 — Suppl Fig. 5 [file 41413_2021_153_MOESM5_ESM.tif]

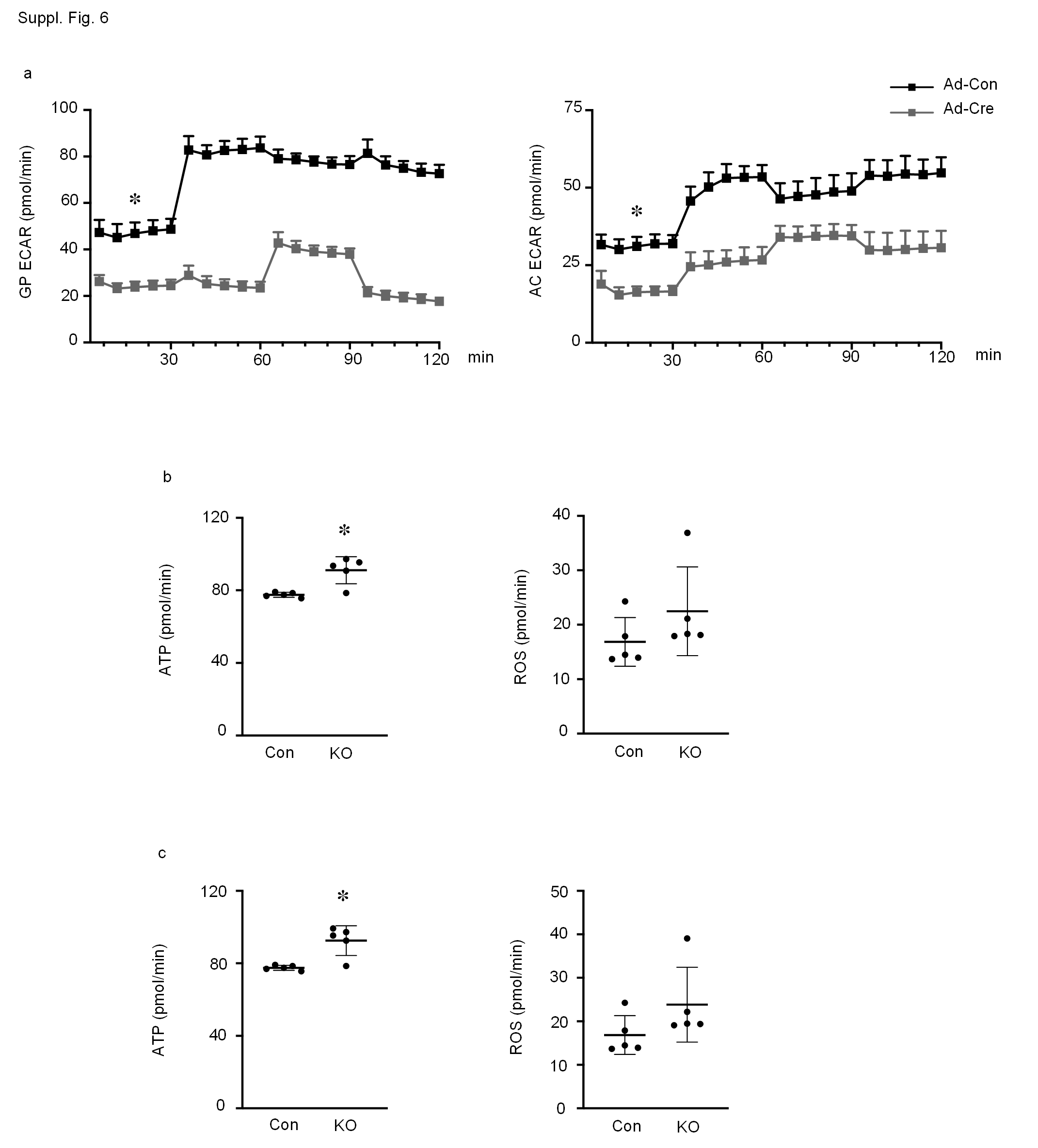

Supplement: Supplementary file 6 — Suppl Fig. 6 [file 41413_2021_153_MOESM6_ESM.tif]
