# Supplementary material for: Deletion of Glut1 in early postnatal cartilage reprograms chondrocytes toward enhanced glutamine oxidation
Source: Bone Res. 2021 Aug 23;9:38. doi: 10.1038/s41413-021-00153-1 (PMC8382841; doi:10.1038/s41413-021-00153-1)
Supplement: Supplementary file 7 — Supplementary information [file 41413_2021_153_MOESM7_ESM.docx]

**Supplementary Figure 1** Agc1Cre^ERT2^ targets both growth plate (GP) and articular cartilage (AC) chondrocytes postnatally. Fluorescence imaging for Ai9 on long bone sections of *Agc1Cre^ERT2^;Ai9^f/+^* mice at 2 months is shown. N=3. Scale bar, 100 μm.

**Supplementary Figure 2** Loss of Glut1 in GP chondrocytes does not affect chondrocyte hypertrophy. **a** Quantification of the Col2a1-positive area in GP tissue according to immunostaining of tibial sections of control and Glut1 LOF mice at 2 months. N=6. **p* <0.05 relative to the controls. **b** Immunostaining for Col10a1 in tibial sections of control and Glut1 LOF mice at 2 months. N=6. Scale bar, 100 μm.

**Supplementary Figure 3** Cartilage remnants persist over time in Glut1 LOF mice. Safranin O/Fast Green staining of tibial sections from 14-month-old control and Glut1 LOF mice. N=4. Scale bar, 100 μm.

**Supplementary Figure 4** Loss of Glut1 in chondrocytes does not affect osteoclast activity in the tibial metaphysis. TRAP staining (**a**) and quantitative analysis of osteoclast activity in the area of bony trabeculae in the tibia metaphyses from 4-month-old (**b**) and 7-month-old (**c**) control and LOF mice. The data are the mean ± SD. N ≥4. Scale bar, 100 μm.

**Supplementary Figure 5** Loss of Glut1 in chondrocytes does not affect bone quality in the tibial metaphysis. The results of micro-CT assessment and quantification of BV/TV of bony trabeculae in the tibial metaphyses from 4- and 7-month-old control and Glut1 LOF mice are shown. The data are the mean ± SD. N=4.

**Supplementary Figure 6** Glycolysis and energy production are markedly altered in Glut1 LOF chondrocytes. **a** A Seahorse assay was used to examine the extracellular acidification rates (ECARs) of control and Glut KO primary GP and AC chondrocytes. The data are the mean ± SD. N=8. **p* <0.05 relative to the controls. **b** ATP and ROS production by control and Glut1 KO primary GP chondrocytes for 24 hours. The data are the mean ± SD. N=5. **p* <0.05 compared to the controls. **c** ATP and ROS production by control and Glut1 KO primary AC chondrocytes for 24 hours. The data are the mean ± SD. N=5. **p* <0.05 compared to the controls.

**Supplemental Table 1. Primer sequences for real-time qPCR**

| Genes | Sequences |
| --- | --- |
| *Glut1* | 5′-CAG TTC GGC TAT AAC ACT GGT G-3′  5′-GCC CCC GAC AGA GAA GAT G-3′ |
| *Glut3* | 5'-AGG TCA CTG AAT TCC TGG GGT-3'  5'-GAA AGA GCC GAT CGT GGC AA-3' |
| *Glut4* | 5'-TCT TAT TGC AGC GCC TGA G-3'  5'-GAG AAT ACA GCT AGG ACC AGT G-3' |
| *Glut5* | 5'-ACA GCT GGC ACT TTG AGG AG-3'  5'-GCG TCA AGG TGA AGG ACT CA-3' |
| *Glut6* | 5'-GGC CTC TGG ATG CTT CTG TT-3'  5'-AGG AGA GTT GGG CAT GAA GC-3' |
| *Glut9* | 5'-CTG AGG AAA GCA CAG GAG GT-3'  5'-GAG CTT CAT GGT CTT GGG CT-3' |
| *Col2a1* | 5'-GCA GAG ATG GAG AAC CTG GTA-3'  5'-AGC CTT CTC GTC ATA CCCT-3' |
| *Acan* | 5'-CGT GTT TCC AAG GAA AAG GA-3'  5'-TGT GCT GAT CAA AGT CCA G-3' |
| *Mmp13* | 5'-AGA CTG GTA ATG GCA TCA AGG-3'  5'-GCC ATT TCA TGC TTC CTG ATG-3' |
| *Adamts5* | 5'-CCT CAC AAC GTC AGT ATA ACC C-3'  5'-GAC GGC ATT ATT GGC TCA AAG-3' |
| *P4ha1* | 5'- AGC CAC CAT TTC AAA CCC AGT-3'  5'-GCC AAG CAC TTT TGC TAA TTC TG-3' |
| *P4ha2* | 5'-CAG GTA CTA TGA TGT GAT GTC CG-3'  5'-AAA GGG TCG CCT TGA GAA GTC-3' |
| *β-actin* | 5'-AGA TGT GGA TCA GCA AGC AG-3'  5'-GCG CAA GTT AGG TTT TGT CA-3' |
